# Exploring an Efficient Remote Biomedical Signal Monitoring Framework for Personal Health in the COVID-19 Pandemic

**DOI:** 10.3390/ijerph18179037

**Published:** 2021-08-27

**Authors:** Zhongyun Tang, Haiyang Hu, Chonghuan Xu, Kaidi Zhao

**Affiliations:** 1 School of Computer Science and Technology, Hangzhou Dianzi University, Hangzhou 310014, China; tangzy@hdu.edu.cn (Z.T.); huhaiyang@hdu.edu.cn (H.H.); 2 School of Information and Electronic Engineering, Zhejiang Gongshang University, Hangzhou 310018, China; 3 School of Business Administration, Zhejiang Gongshang University, Hangzhou 310018, China; 4 Modern Business Research Center, Zhejiang Gongshang University, Hangzhou 310018, China; 5 Zheshang Research Institute, Zhejiang Gongshang University, Hangzhou 310018, China; 6 School of Information Science and Technology, Fudan University, Shanghai 200433, China; 19110720083@fudan.edu.cn

**Keywords:** telemedicine, biomedical signal monitoring framework, GRU-AE, COVID-19 pandemic, healthy monitoring

## Abstract

Nowadays people are mostly focused on their work while ignoring their health which in turn is creating a drastic effect on their health in the long run. Remote health monitoring through telemedicine can help people discover potential health threats in time. In the COVID-19 pandemic, remote health monitoring can help obtain and analyze biomedical signals including human body temperature without direct body contact. This technique is of great significance to achieve safe and efficient health monitoring in the COVID-19 pandemic. Existing remote biomedical signal monitoring methods cannot effectively analyze the time series data. This paper designs a remote biomedical signal monitoring framework combining the Internet of Things (IoT), 5G communication and artificial intelligence techniques. In the constructed framework, IoT devices are used to collect biomedical signals at the perception layer. Subsequently, the biomedical signals are transmitted through the 5G network to the cloud server where the GRU-AE deep learning model is deployed. It is noteworthy that the proposed GRU-AE model can analyze multi-dimensional biomedical signals in time series. Finally, this paper conducts a 24-week monitoring experiment for 2000 subjects of different ages to obtain real data. Compared with the traditional biomedical signal monitoring method based on the AutoEncoder model, the GRU-AE model has better performance. The research has an important role in promoting the development of biomedical signal monitoring techniques, which can be effectively applied to some kinds of remote health monitoring scenario.

## 1. Introduction

Telemedicine is the use of electronic information and telecommunication technology to provide the health care that people need while practicing social distancing [1]. The fields of application of telemedicine are various and constantly evolving, from cardiology (transmission of electrocardiographic traces) to radiology (transmission of radiographic images), dermatology (transmission of digital images of skin lesions), pathological anatomy, and many others. Due to the COVID-19 pandemic that has swept the world, health monitoring services in telemedicine have developed explosively [2]. The remote health monitoring services mainly diagnose and evaluate health issues by monitoring various biomedical signals. A health issue occurs when the normal metabolism of the body fails or is altered due to pollutants, pathogens or other means that cause health problems that are considered as diseases. Common biomedical signal parameters include: pulse, body temperature, blood oxygen saturation concentration, blood glucose concentration and blood pressure [3]. With the popularization of health monitoring equipment, many non-medical professional equipment such as smart bracelets are widely favored by people. The equipment can provide users with the display of various biomedical signals through real-time monitoring while lacking an analytical function [4,5]. Even professional telemedicine monitoring equipment cannot perform the comprehensive analysis of multi-dimensional biomedical signal data in time series, and quickly find potential health issues. As we all know, the COVID-19 pandemic has been growing rapidly because of a new variant, which is also a serious problem in many countries. Therefore, the demand for a remote health monitoring service is more urgent for measuring the body temperature aspect. In the meantime, this requires higher requirements for the intelligence gathering and accuracy of remote health monitoring. For example, the occasional rise in body temperature may be caused by exercise or stress, rather than a precursor to infection with COVID-19.

Nowadays, remote health monitoring relies on artificial intelligence, the Internet of Things (IoT), cloud computing and other Internet technologies [6]. There are many research works focused on how to obtain all kinds of biomedical signals through integrated technologies. For example, in the aspect of biomedical signal monitoring, biomedical signal data can be obtained through wearable IoT sensor devices. These data are transmitted to the cloud server through a high-speed network for subsequent processing and analysis. Finally, the potential health issues can be sent to users or medical staff [7]. These methods provide important support for the realization of intelligence, real-time and accuracy of remote health monitoring, but still have some shortcomings. A few methods can provide intelligent single biomedical signal analysis while they lose efficacy when facing multiple biomedical signals. Some others fuse multi-dimensional physiological signals, but ignore the time influence.

In order to solve the problems of existing remote biomedical signal monitoring methods, this paper introduces the IoT, 5G communication, artificial intelligence and other technologies and constructs a biomedical signal monitoring framework based on GRU-AE deep learning model, which realizes the automatic collection and intelligent analysis of multi-dimensional biomedical signals. The framework collects biomedical signals through IoT sensing devices, and then uses high-speed mobile communication networks to transmit them to the cloud server in real time. The GRU-AE model deals with the correlated biomedical signals based on time series to achieve more accurate and efficient intelligent health monitoring. The proposed framework has the strengths of fast response and high accuracy. It is especially suitable for the intelligent monitoring of biomedical signals such as body temperature under the COVID-19 pandemic.

The structure of this paper is organized as follows. In Section 2, related work is introduced. In Section 3, The biomedical signal monitoring framework based on deep learning is elaborated, including its application scenarios and workflow. Section 4 introduces the basic structure of the GRU-AE model and the principle that it is used to process and monitor multi-dimensional biomedical signals in time series. Section 5 reveals experimental and analytical process on two biomedical signals and indicates the performance of our proposed model. Finally, we draw conclusions in Section 6.

## 2. Related Works

In recent years, many remote health monitoring methods based on multi-techniques have emerged. Most monitoring frameworks are implemented using technologies such as the IoT and wireless sensor networks. Yang [8] designed a multi-sensor bracelet to collect physical information and uploaded it to a cloud server through the IoT. After extracting the features of the experimental data, an evaluation model of these biological characteristics and human health emotions was established, and the mapping relationship between multi-sensor data and mental conditions was mined. Xing [9] designed a set of wearable devices that could collect the ECG signals of patients with cardiovascular diseases. The data were transmitted to the self-built cloud server through the IoT, which could store the complete ECG signal data and display it on the web in real time. It implemented a system for monitoring electrical signals through the IoT. Zubair et al. [10] designed an economical, efficient, and low-power wearable smart band based on IoT. This band detected mental stress based on skin conductance and was mainly used for medical care. It could also continuously monitor the user’s mental stress, and wirelessly transmitted stress-related data to the user’s smartphone. Isabel et al. [11] designed a pressure monitoring system using wearable devices based on IoT. This system included a smart bracelet module and a chest strap module, which could be worn on the wrist and chest respectively. The parameters of system monitoring such as electrical skin activity and heart rate in real time were sent to a cloud-based server. And The server was used as an online IoT platform, using applications to perform data calculations and displayed stress reports. Sravanthi and Ganesan [12] proposed an IoT-based health monitoring device that tracked the heart rate and body heat and also sent an email/SMS warning when these readings went above the main values. Thingspeak and Google sheets logged heart rate and body temperature measurements so that patient wellbeing could be tracked on the internet everywhere in the world. There would also be a rush so that patients could quickly send messages to their families. Imberti et al. [13] found that device-based remote monitoring (RM) was useful in the early detection of cardiac implantable electronic devices (CIEDs) technical issues and cardiac arrhythmias. Moreover, RM allowed the continuous monitoring of several patients’ clinical parameters associated with impending heart failure (HF) decompensation, but there was still uncertainty regarding its effectiveness in reducing mortality and hospitalizations. Charrad et al. [14] designed a monitoring system for heart patients. This system was called ECG Patch Monitor, which could collect and analyze heart data in an actual environment (for example, at work or at home). Once an abnormality was detected, the device would alert the medical center. Subsequently, the medical center remotely controlled the ECG patch monitor through the platform.

Furthermore, artificial intelligence technology has also been widely used in the fields of medical health and biomedical signal processing [15]. Guo [16] autonomously learned the spatial and temporal characteristics of ECG by constructing a CNN-LSTM hybrid model. He utilized multi-task learning to classify diseases. Finally, an ECG monitoring system was realized based on a deep learning model, which included the functions of ECG signal data acquisition and analysis. Liu et al. [17] analyzed different types of ECG and used the support vector machine method to identify arrhythmia. The proposed method could effectively identify four types of arrhythmia biometric signals. Zhang et al. [18] trained an LSTM deep learning model through a large number of biometric signals in the physical examination records of pregnant women. Finally, the prediction of fetal weight was realized, and experiments proved that the model achieved good prediction effect on abnormal weight. Sun et al. [19] proposed a CNN-based method for doctors to identify potential risks through biometric identification analysis. This method automatically learned features from the original biometric signal and identified abnormal monitoring of physiological signals through unsupervised learning and multivariate Gaussian distribution. The experimental results proved that the method could be applied to identify early signs of illness. Paraschiakos et al. [20] proposed an architecture consisting of an RNN with 3 GRU layers and a feedforward network combining both accelerometer and participant-level data. It could measure physical activity energy expenditure for older people. Xiao [21] designed a deep ensemble network of CNN and GRU for time-series classification. It could be applied to ICU time series data and combined with static data to achieve high-performance intubation prediction. Sharma et al. [22] proposed a model based on IoT for early detection of COVID-19 based on ontology method using sensory 1D biomedical signals such as ECG, PPG, temperature, and accelerometer. This model extracts the characteristics of the four biological signals and uses the MDCA method for fusion, and finally uses SVM and KNN for classification, which realizes the classification and monitoring of abnormal biological signals. Most of the applications of artificial intelligence in the medical field are image processing based on deep learning [23,24]. It is applied to medical physiological images, such as gesture recognition, electrocardiogram, ultrasound image classification and prediction [25]. There are few studies on the use of deep learning anomaly monitoring methods in biomedical signal monitoring.

In summary, artificial intelligence, IoT and other techniques have been applied in the field of biomedical signal monitoring and medical health. However, the current related research works have some limitations. Remote healthy monitoring through the IoT and cloud computing is simple collection. Biomedical signals are simply recorded or reminded that they exceed the normal range. Most biomedical signal processing methods lack analysis of biomedical signal data in time series. They cannot process multi-dimensional biomedical signal data in parallel [26]. At present, there are few research works on realizing remote intelligent health monitoring by combining technologies such as the IoT, cloud computing, and artificial intelligence to automatically collect, process and analyze biomedical signals. In particular, the research results of remote health monitoring for the prevention and control of the COVID-19 pandemic are relatively insufficient [27].

## 3. Biomedical Signal Monitoring Framework Based on Deep Learning

**Figure 1:**
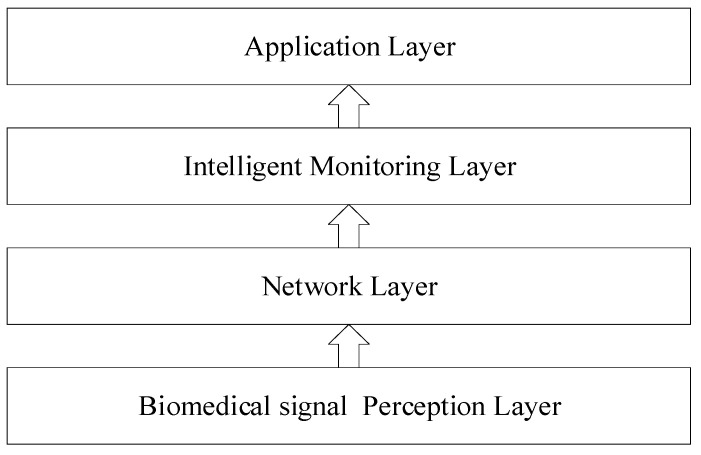
Intelligent biomedical signal monitoring framework.

The proposed biomedical signal monitoring framework consists of four layers including: (1) Biomedical signal perception layer, (2) Network layer, (3) Intelligent monitoring layer and (4) Application layer, shown in Figure 1.

(1) The biomedical signal perception layer is the sensing device layer. In this layer, IoT devices (i.e., body temperature monitoring device, heart rate monitoring device, accelerometers, finger imaging) are widely used to collect different biomedical signals from users.
(2) The network layer is responsible for transmitting the data collected by perception layer. The network connection between this layer and the perception layer usually relies on a wireless sensor network, such as Wi-Fi, Bluetooth, etc. The data transmission is based on various communication networks such as LAN, wired broadband and mobile communication networks.
(3) The intelligent monitoring layer is the most critical part of this framework. The deep learning model is deployed here. It generally has three main steps: (1) data processing, (2) deep learning model training, (3) real-time monitoring. This layer is generally deployed in the cloud. It can even be deployed in the edge cloud [28]. In the experiment of this paper, 5G communication network [29] is used at the network layer simultaneously, this is the perfect solution for the entire framework.
(4) The application layer is used to deploy applications suitable for this framework. At present, the main application proposed in this paper is health monitoring. It can also provide some other health data statistical analysis applications for biomedical signal data, such as health state assessment and prediction.

**Figure 2:**
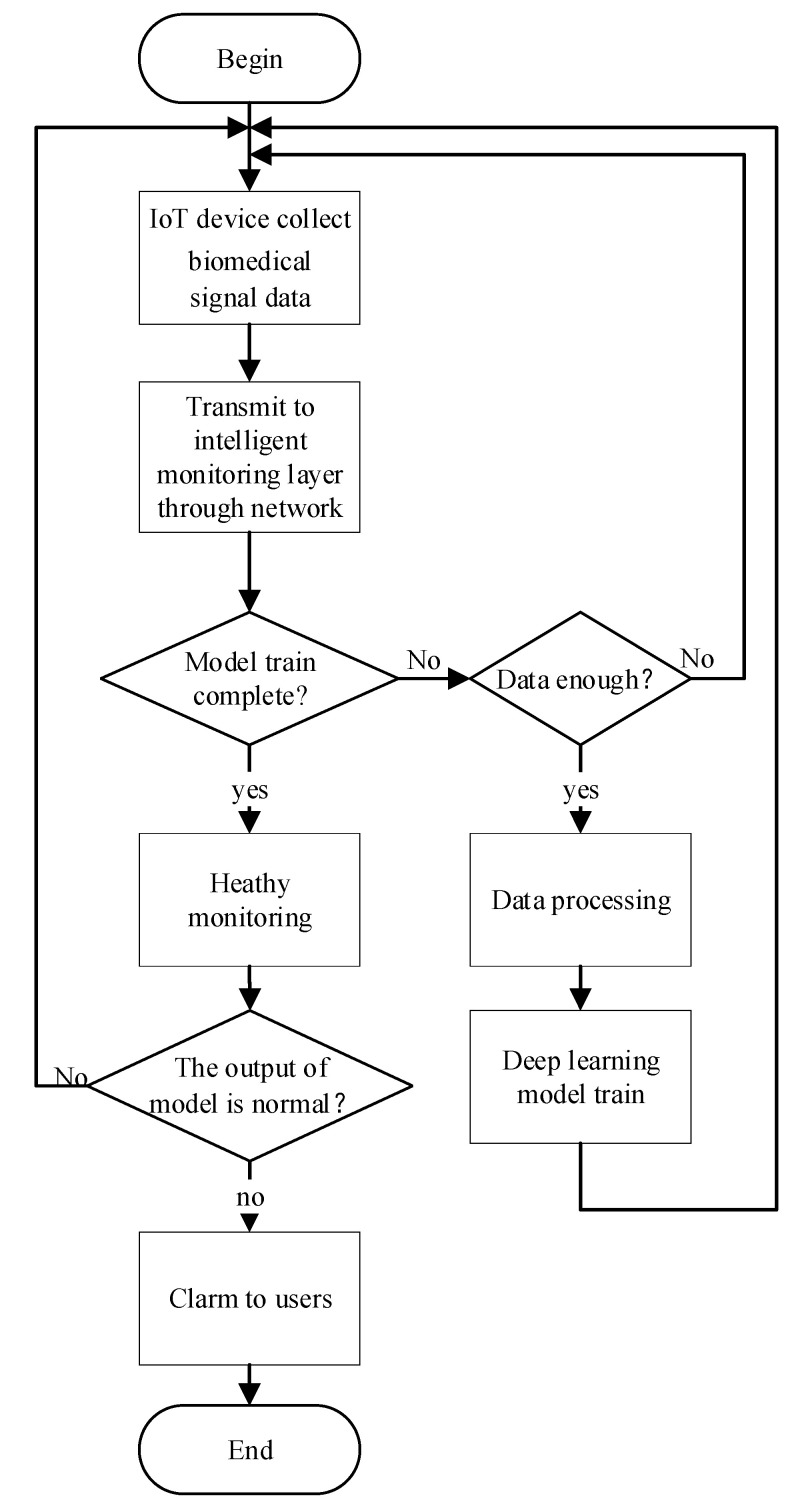
The workflow of biomedical signal intelligent real-time monitoring.

The workflow of the entire framework is shown in Figure 2. Firstly, the biomedical signal perception layer collects biomedical signals at any time through IoT devices. The data are transmitted to the intelligent monitoring layer through the network layer. Secondly, the intelligent monitoring layer preprocesses the collected data. When the collected data reach the threshold for the training set, the intelligent monitoring layer starts model training. When the model training is completed, the biomedical signals collected from the perception layer will be input into the model for health monitoring. If the output result is abnormal, it means that health issues may occur. An alarm will be sent to the user.

**Figure 3:**
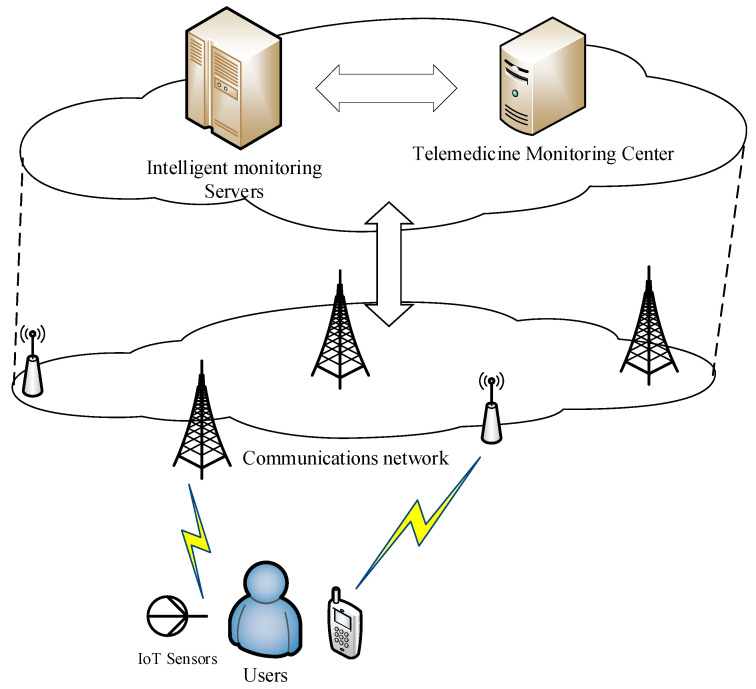
Actual application scenario of the biomedical signal monitoring framework.

The actual application scenario of the biomedical signal monitoring framework is shown in Figure 3. The wearable IoT sensor device collects users’ biomedical signal data and transmits them through the fast communication networks (i.e., 5G communication network). Deep learning models are deployed in cloud servers (such as edge computing servers). These models are capable of connecting multiple users and establish the remote transmission of biomedical signal data. Finally, the monitoring results will continue to be sent to users or telemedicine health centers. In the COVID-19 pandemic scenario, the entire framework can remotely collect the body temperature data of a large number of quarantined persons, and establish models for intelligent monitoring. The monitoring results can be sent to users and the pandemic prevention and control center in real time. The prevention and control center can grasp the temperature changes or other health issues in real time without direct contact, so as to make accurate prevention and control decisions.

## 4. Intelligent Biomedical Signal Monitoring Model

The intelligent biomedical signal monitoring model is deployed in the intelligent monitoring layer [30]. Biomedical signal health monitoring is related to time. Therefore, deep learning models which can deal with the time series data are generally used. For example, recurrent neural network (RNN) is a type of artificial neural network which uses sequential data or time series data. However, the problem of gradient disappearance and gradient explosion during long sequence training may occur in RNN. LSTM is a variant model of RNN to solve the above problems. Furthermore, a simplified version of LSTM named GRU proposed by Cho, et al. [31] is widely used to address the ‘short-term memory’ issue plaguing vanilla RNNs. GRU is able to effectively retain long-term dependencies in sequential data. This paper proposes a model combining GRU and AutoEncoder to realize biomedical signal real-time monitoring.

### 4.1. Introduction of Monitoring Model

#### 4.1.1. AutoEncoder

**Figure 4:**
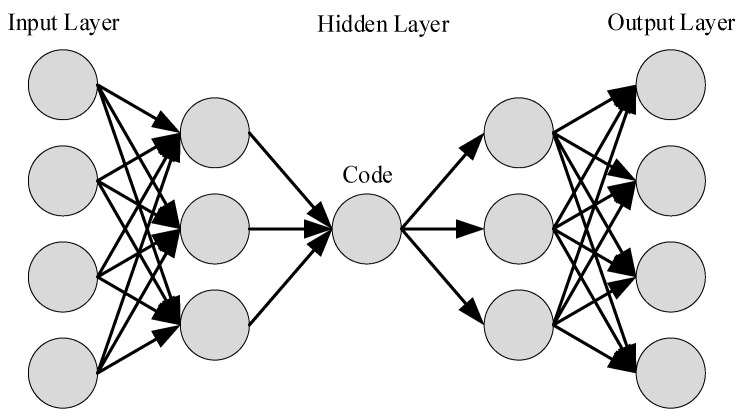
AutoEncoder model.

AutoEncoder is an unsupervised artificial neural network that is used to efficiently compress and encode data to achieve the goal of dimensionality reduction. The schematic diagram of AutoEncoder model is shown in Figure 4.

AutoEncoder consists of three layers including an input layer, a hidden layer, and the output layer. Input and output layers have an equal number of nodes in AutoEncoder. This is because the purpose of the AutoEncoder is to initialize the hidden layer parameters that will reconstruct the multidimensional input data. Encoding is the process between the input layer and the hidden layer. The encoder is usually used to reduce multidimensional data to low-dimensional data (compressed representation), whereas it may allow different representations of equal dimension and higher dimensional data (sparse representation) to be obtained. The decoding constructs the process of the output using hidden layer output weights between the hidden layer and the output layer. Decoding aims to reconstruct the input data by using the sparse or compressed representations into preferred dimensionality. 

In monitoring applications, the output data need to be as close to the original data as possible. The monitoring function is realized by evaluating the difference between the output data and the original data. The encoding and decoding process are shown in the following formulas.

(1) y=f(Ax+b)

(2) $$x^{\prime }=g\left(Cy+d\right)$$

The objective function is when the difference between input and output is the smallest. It means the loss is the smallest *l*. It is shown in the following formula.

(3) $$l=argmin_{y,x^{\prime }}Loss$$

(4) $$Loss\left(x,x^{\prime }\right)={\left\|x-x^{\prime }\right\|}^{2}$$

(5) $$Loss\left(x,x^{\prime }\right)=\|x-g{\left(Cf\left(Ax+b\right)+d)\right\|}^{2}$$

After the model has been trained, if the Loss exceed the threshold, it indicates that the data is abnormal.

#### 4.1.2. LSTM

**Figure 5:**
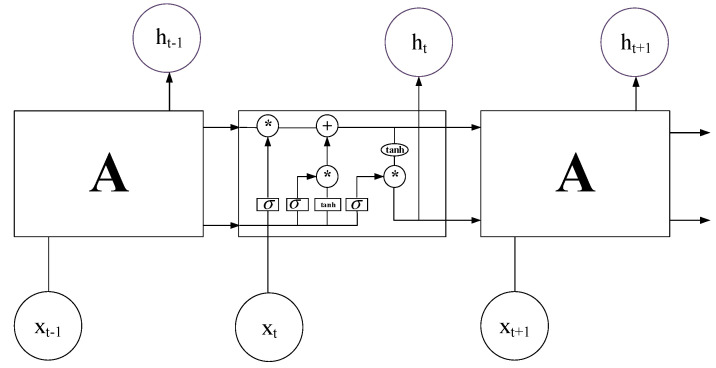
LSTM model. ‘*’ represents convolution operation.

LSTM has a complex structure, and the model is shown in Figure 5. The selective power of LSTM is archived by the gate state which helps remember information that needs long-term memory and opt out unimportant information by controlling the information transmission [32].

In addition to the sample feature *x* and the hidden layer output $h_{t-1}$ of the previous time, the LSTM input also adds memory cell $c_{t-1}$. These inputs need to pass through the input gate $i_{t}$, the forget gate $f_{t}$, the output gate $o_{t}$ and the candidate memory cell $\tilde{c_{t}}$ inside the structure. $c_{t}$ denotes the cell state for time t. It is yielded from the previous cell state $c_{t-1}$ plus some values. It is mainly used to save the data passed by the previous unit. The information passed each time will be ‘forgotten’ in certain dimensions, and the information contained in the current node will be added. Therefore the value of $c_{t}$ changes are relatively small. $h_{t}$ is mainly for combining with the current input to obtain the gate signal. For different current inputs, the $h_{t}$ passed to the next state will differ greatly. The related calculation formula is as follows.

(6) $$\{\begin{array}{c} i_{t}=\sigma \left(W\left[h_{t-1},x_{t}\right]+b_{i}\right)\\ f_{t}=\sigma \left(W\left[h_{t-1},x_{t}\right]+b_{f}\right)\\ \begin{array}{c} o_{t}=\sigma \left(W\left[h_{t-1},x_{t}\right]+b_{o}\right)\\ \tilde{c_{t}}=\tan h\left(W\left[h_{t-1},x_{t}\right]+b_{o}\right) \end{array} \end{array}$$

The update formula of the memory cell is as follows.

(7) $$c_{t}=f_{t}*c_{t-1}+i_{t}*\tilde{c_{t}}$$

The update formula of the hidden cell is as follows.

(8) $$h_{t}=o_{t}*\tan h\left(c_{t}\right)$$

The final output y is calculated by the deformation of the h.

(9) $$\hat{y_{t}}=\sigma \left(W_{y}h_{y}+b_{y}\right)$$

Among them, the excitation function σ is the sigmoid function.

#### 4.1.3. GRU

GRU is mainly proposed to solve the problem of gradient disappearance in standard recurrent neural networks. Since GRU and LSTM are very similar, GRU can also be regarded as a variant of LSTM. The GRU only has an update gate and a reset gate. These two gate vectors determine which information should be passed to the output. Therefore, there are fewer parameters and calculations in GRU. It is easier to converge and has higher efficiency. However, when the data set is large, LSTM expression performance is better.

**Figure 6:**
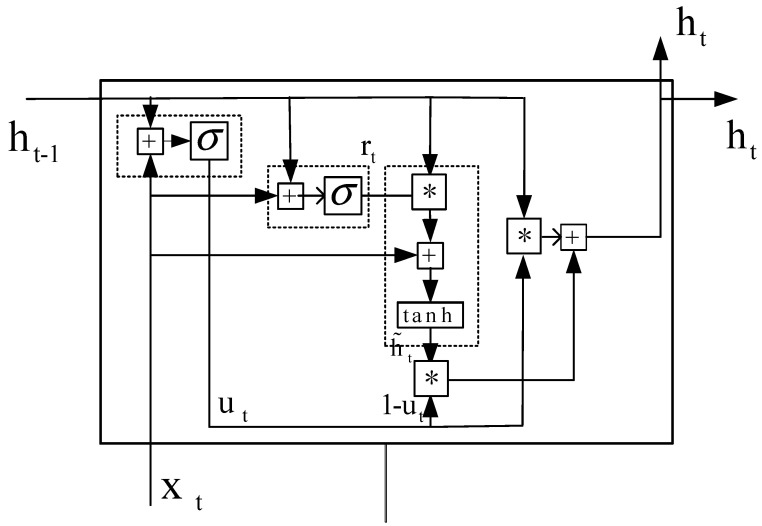
Unit of GRU model. ‘*’ represents convolution operation.

In order to describe the GRU message transfer process in detail, the specific GRU unit structure is shown in Figure 6. *σ* represents the sigmoid function. $u_{t}$ represents the update function, $x_{t}$ represents the input, $h_{t}$ represents the output,$\;r_{t}$ represents the reset function, and ${\tilde{h}}_{t}$ represents the current memory content. The role of the update gate is to help the model to convey the amount of past information. In this way, the model can solve the problem of gradient messages.

The GRU model can use the reset gate to store past information. Firstly, the product of the input $x_{t}$ and the weight *W*, the product of $h_{t-1}$ and the weight *U* should be obtained. Then the product of reset gate $\;r_{t}$ and $h_{t-1}$ will be obtained element by element. Finally, using the tanh function to find the memory content ${\tilde{h}}_{t}$. In the last step of the GRU unit, $h_{t}$ needs to be calculated, which saves the information of the current unit and transmits it to the network. GRU uses the update gate to determine the information read from the memory content and the previous unit. The calculation process is shown in formula (10) to formula (13).

(10) $$u_{t}=\sigma \left(W^{u}x_{t}+U^{u}h_{t-1}\right)$$

(11) $$r_{t}=\sigma (W^{r}x_{t}+U^{r}h_{t-1})$$

(12) $${\tilde{h}}_{t}=\tan h\left(Wx_{t}+r_{t}*Uh_{t-1}\right)$$

(13) $$h_{t}=u_{t}*h_{t-1}+\left(1-u_{t}\right)*{\tilde{h}}_{t}$$

### 4.2. GRU-AE Monitoring Model

**Figure 7:**
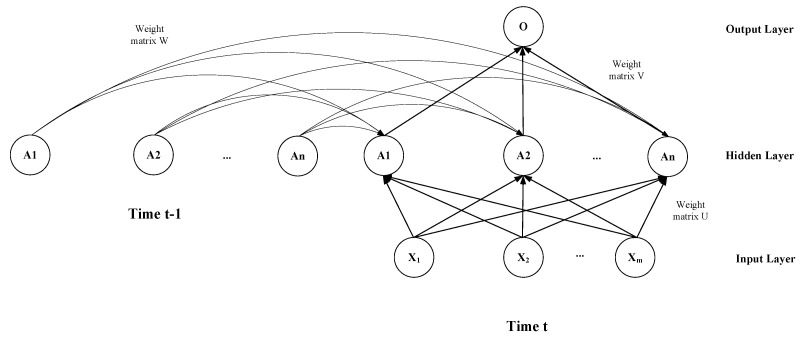
Time series learning model.

Both GRU and LSTM are models related to time series. The state of the previous moment is used as the input of the current moment. The memory is selected according to the gate. The state of the model in time is shown in Figure 7. We let set $\left\{X_{1},X_{2},\cdots ,X_{M}\right\}$ indicate *m* types of biomedical signal data. Each unit represents a single LSTM unit or GRU unit in Figure 4 and Figure 5. The model has an output result at each moment and inputs it to the next moment.

**Figure 8:**
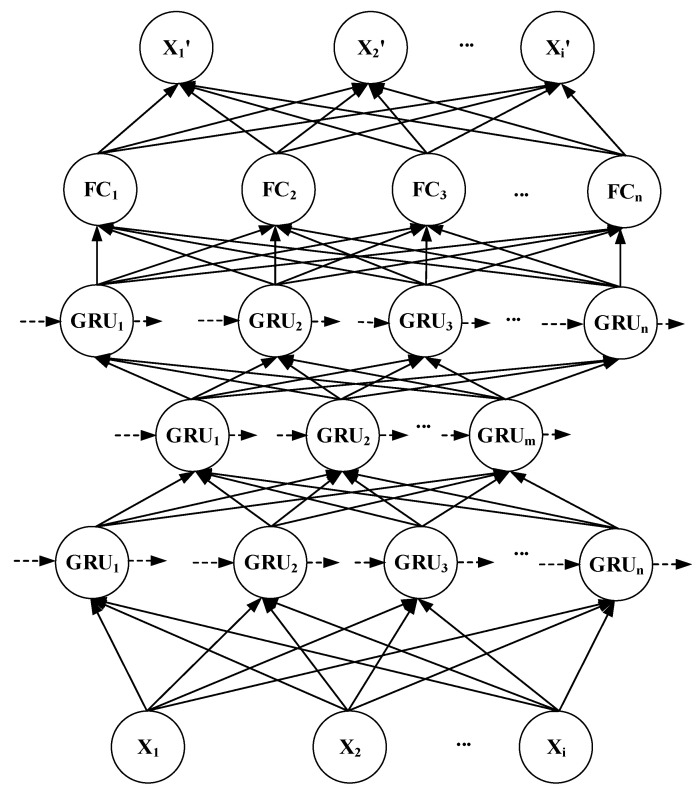
GRU-AE model.

Biomedical signal data monitoring must meet the requirements of both monitoring function and time series. This paper combines the AutoEncoder model and the GRU model, using the GRU unit as the node of the AutoEncoder. It constructs a three-layer GRU-AutoEncoder (GRU-AE) model. A fully connected layer is added to the output layer. This can make the dimensionality of the output data consistent with the input data. The entire model is shown in Figure 8.

The input data of the model is biomedical signal data. The feature dimension of the input data is determined by the classes of the biomedical signals. The first layer of the model is the same as the ordinary GRU model. The number of GRU units is reduced in the second layer. The output of the previous layer is used as the input of this layer. The third layer has the same number of units as the first layer to achieve decoding. At the same time, each layer takes $h_{t-1}$ as input, which from the previous moment. The output $h_{t}$ of the model at the current moment will also be used as the input at the next moment. Finally, to calculate the loss, the output of the entire model is kept consistent with the dimensions of the input. A fully connected layer is added to the fourth layer. The input dimension is consistent with the dimension of the last layer. The output dimension is consistent with the input dimension.

The input of the first layer is that the collected biomedical signal data are $x_{t}$. This represents the data at time *t*, which are composed of *n* kinds of biomedical signal, as shown in the formula (14).

(14) $$x_{t}=\left\{X_{i}|i=1\ldots n\right\}$$

Each GRU unit has two inputs and one output, which is a function of binary independent variables. The *k*-th layer is represented by the superscript on the right. Then, the relationship between the input and output of the first layer at time t is:

(15) $$h_{t}^{1}=g^{1}\left(x_{t},h_{t-1}^{1}\right)$$

The function *g* represents a series of function combinations of the GRU model introduced earlier.

The input of the second layer becomes the output of the previous layer. The input and output relationships of the second and third layers are respectively:

(16) $$h_{t}^{2}=g^{2}\left(h_{t}^{1},h_{t-1}^{2}\right)$$

(17) $$h_{t}^{3}=g^{3}\left(h_{t}^{2},h_{t-1}^{3}\right)$$

After going through the fully connected layer:

(18) $$y_{t}=\sigma \left(W_{y}h_{t}^{3}+b_{y}\right)$$

In general, supervised learning is to find the minimum value of the objective function MSE, as shown in formula (19). Its role is to make the output of the model approximate the label value.

(19) $$Loss=\frac{1}{n}\sum_{i=1}^{n}{\left(y_{i}-\hat{y_{i}}\right)}^{2}$$

The Autoencoder is to find the minimum loss between output and input. Its goal is to make the output as close to the input as possible. The loss function at a certain time *t* is:

(20) $$Loss_{t}=\frac{1}{2}{\left(y_{t}-x_{t}\right)}^{2}$$

The loss at all moments is:

(21) $$Loss=\sum_{t=1}^{T}\frac{1}{2}{\left(y_{t}-x_{t}\right)}^{2}$$

(22) $$Loss=\sum_{t=1}^{T}\frac{1}{2}{\left(\sigma \left(W_{y}\left(g^{3}\left(g^{2}\left(g^{1}\left(x_{t},h_{t-1}^{1}\right),h_{t-1}^{2}\right),h_{t-1}^{3}\right)\right)+b_{y}\right)-x_{t}\right)}^{2}$$

Therefore, the ultimate goal is to make loss tend to 0. Each parameter is obtained by the back propagation principle of a neural network. According to the gradient descent iterative update principle of back propagation, combining the input-related functions in back propagation, the update of some parameters in the coding layer is shown in the following formula:

(23) $$\frac{\partial Loss}{\partial W}=h_{t}^{2^{T}}\frac{\partial Loss}{\partial {\tilde{h}}_{t}}$$

(24) $$\frac{\partial Loss}{\partial W^{r}}=h_{t}^{2^{T}}\frac{\partial Loss}{\partial {\tilde{r}}_{t}}$$

(25) $$\frac{\partial Loss}{\partial W^{u}}=h_{t}^{2^{T}}\frac{\partial Loss}{\partial {\tilde{u}}_{t}}$$

Through the GRU-AE model, multiple biomedical signals can be correlated in time series. The model uses a variety of biomedical signals and historical states as the basis for monitoring health. Such standards are more scientific and accurate than other monitoring models, and even human judgments. In addition, the GRU-AE model only needs biomedical signals of the health state for training.

**Figure 9:**
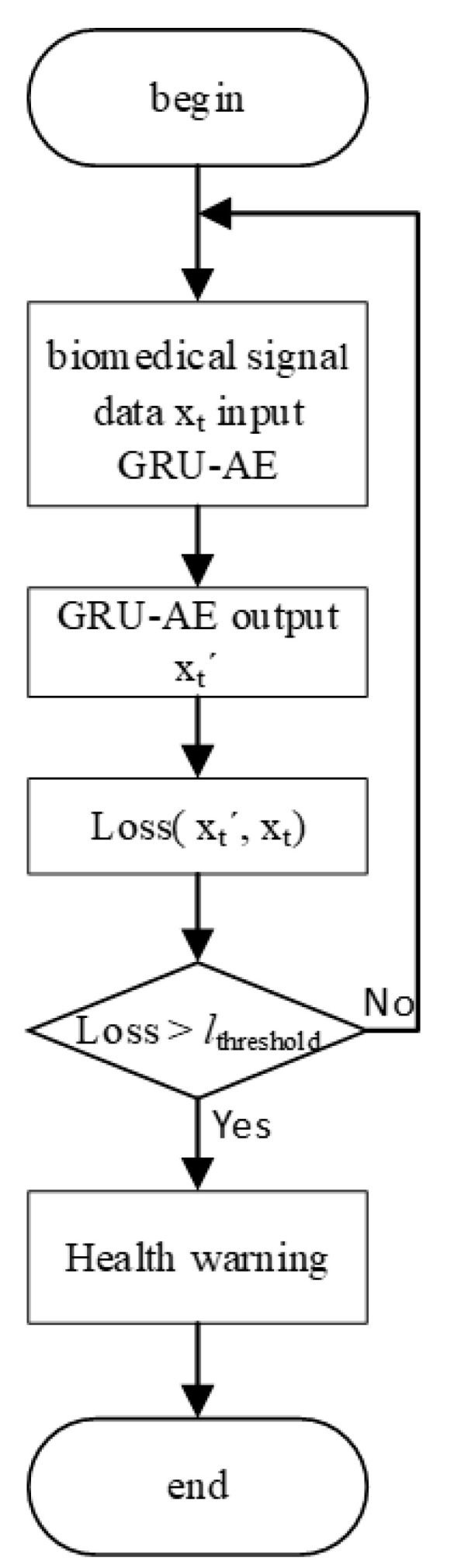
Workflow of monitoring.

After the GRU-AE model has been trained, it can start to monitor health. The flowchart of the monitoring process is shown in Figure 9. The biomedical signal data are processed to satisfy the input format of the model. After inputting the biomedical signal data into GRU-AE model, output data will be obtained. Loss value is derived from the difference between the output data and the original biomedical signal data. If the loss value does not exceed the threshold, the biomedical signal is normal and there are no health issues. The model continues to monitor the next moment of the input biomedical data. If the loss value exceeds the threshold, it indicates that the output has a large deviation from the original data which in turn indicates the biomedical signal is abnormal, and a health issue may occur. At this time, the health warning is sent to the client or the monitoring center. 

### 4.3. Experimental Data

The experimental platform built in this paper collected a variety of biomedical signals from the subjects, and analyzed them with heart rate and body temperature as examples. In the COVID-19 pandemic prevention and control scenario, body temperature is the main monitored parameter. When there is an issue with human health, body temperature and heart rate are usually abnormal, rising or falling. Doctors often judge whether there are health issues according to the abnormal body temperature and heart rate [33]. However, there are also some special cases, such as bed rest and sedentary, where heart rate will decrease. During sports, such as running, heart rate and body temperature rise.

In answering to the feasibility and credibility of the experiment, this study selected subjects of different age and gender who could be easily tracked. This paper selects 2000 subjects between the ages of 18–50 and the duration is 24 weeks. Specifically, there were 978 male and 1022 female who are evenly distributed in the range of 18–50. The males accounted for 48.9% of the total subjects. In addition, all 2000 subjects had no special diseases and were healthy in the month before the tracking test.

In this experiment, a variety of biomedical signals were collected by wearable IoT sensor devices. Biomedical signal data were collected every hour, and a total of 8,064,000 sets of biomedical signal data were obtained. Among them, 613 subjects had health issues (i.e., based on the diagnosis given by the doctor) during the experiment period.

In order to verify the performance of the model, this paper divided the acquired data into a train set and a test set. More specifically, based on the time series characteristics of biomedical signals, we selected the data from week 1 to week 20 as the training set, including 2000 subjects containing 6,720,000 data. The data from week 21 to week 24 were the test set, including 2000 subjects containing 1,344,000 data. For each subject, there were a total of 672 sets of biomedical signal data in the 4 weeks of the test set. 

In this paper, a 20-year-old male subject and a 50-year-old female subject are taken as examples to analyze and elaborate the experimental results. The data of the former are defined as test set 1, and the data of the latter are defined as test set 2, shown in Table 1. 

### 4.4. Evaluation Metrics

The mean absolute error (*MAE*) measures the average magnitude of the errors in a set of forecasts, without considering their direction. It measures accuracy for continuous variables. The root mean squared error (*RMSE*) is a quadratic scoring rule which measures the average magnitude of the error. The *MAE* and the *RMSE* can be used together to diagnose the variation in the errors in a set of forecasts. The formulas of *MAE* and *RMSE* can be expressed as follows.

(26) $$MAE=\frac{1}{m}\sum_{i=1}^{m}\left|y_{i}-{\hat{y}}_{i}\right|$$

(27) $$RMSE=\sqrt{\frac{1}{m}\overset{m}{\underset{i=1}{\sum }}{\left(y_{i}-{\hat{y}}_{i}\right)}^{2}}$$

The *RMSE* will always be larger or equal to the *MAE*; the greater difference between them, the greater the variance in the individual errors in the sample. If the *RMSE* = *MAE*, then all the errors are of the same magnitude [34,35]. 

When the test set data are input to the trained model, if the output result is very close to the input, it means that the data are a healthy biomedical signal. When the model output differs greatly from the input, it means that biomedical signal is unhealthy. Therefore, the difference can be used to determine whether there is an unhealthy state. The size of the threshold determines the performance of the model. Similar to most classification models, this paper evaluates the accuracy of model by constructing a confusion matrix. *TP*, *TN*, *FP*, *FN* respectively represent the number monitored to be healthy and actually healthy, the number monitored to be unhealthy and actually unhealthy, the number monitored to be healthy and actually unhealthy, and the number monitored to be unhealthy and actually healthy. Then the true positive rate (*TPR*) is shown in the following formula:

(28) $$TPR=\frac{TP}{TP+FN}$$

The false positive rate (*FPR*) is shown in the following formula:

(29) $$FPR=\frac{FP}{FP+TN}$$

The accuracy is defined by the following formula:

(30) $$accuracy=\frac{TP+TN}{TP+FN+FP+TN}$$

The threshold is different, the value of *TPR*, *FPR* and accuracy will change. These evaluation methods are widely used in information retrieval, recommender systems and other fields [36,37,38,39,40]. 

In the case of changing the threshold, the ROC curve of the relationship between *TPR* and *FPR* is constructed to evaluate the effect of the model. A positive sample and a negative sample are randomly selected. The probability that the model predicts that the probability of the positive sample is positive is greater than the value of the probability that the model predicts the negative sample to be positive is the value of *AUC*. The *AUC* calculation formula is as follows:

(31) $$AUC=\frac{\sum_{i\epsilon positiveclass}rank_{i}-\frac{M\times \left(M+1\right)}{2}}{M\times N}$$

Therefore, *AUC* is the area under the ROC curve. The larger its value, the better the model performance. 

## 5. Experiment

The experimental platform of this paper was composed of IoT sensing equipment, a wireless router supporting 5G communication, and a high-performance cloud server. The IoT sensing device was connected to the router through Wi-Fi, and the router transmitted data to the server through the 5G network. The GRU-AE monitoring model of this paper was deployed in the server. The free version of PyCharm community was used as the development environment on the Ubuntu 16.04 operating system. All methods were implemented in Python.

### 5.1. Parameters Determination

This paper compares GRU-AE model with the AutoEncoder model with an identical parameter setting. They are three-layer neural networks. The first layer has 128 neural units, the second layer has 32 neural units, and the third layer also has 128 neural units. The fully connected output layer is a transformation of 128-dimensional data into two-dimensional data consistent with the input. The learning rate of the model is 0.01, and the epochs of model training are 4000.

### 5.2. Experimental Results

**Figure 10:**
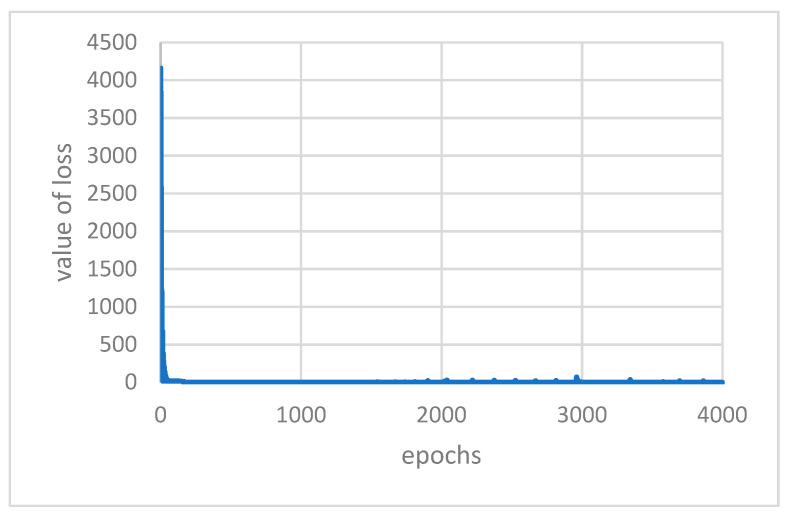
Value of loss with epochs in AutoEncoder.

**Figure 11:**
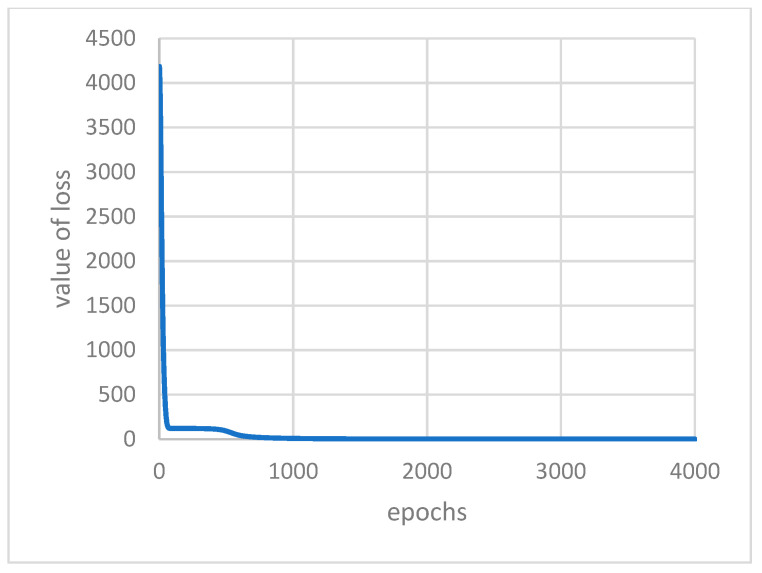
Value of loss with epochs in GRU-AE.

Under the above conditions of determining the parameters and train set firstly, these two models were trained. Figure 10 and Figure 11 display the loss function varies with the epochs of iterations of AutoEncoder model and GRU-AE model, respectively. It can be found from the figures that the convergence effect of the two loss functions is good. The parameters and calculation amount of AutoEncoder are relatively small, and the convergence speed is faster.

**Figure 12:**
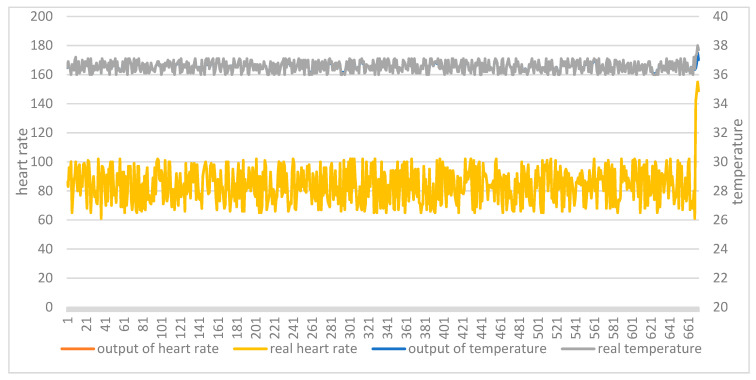
Comparison of input and output of AutoEncoder model in test set 1.

**Figure 13:**
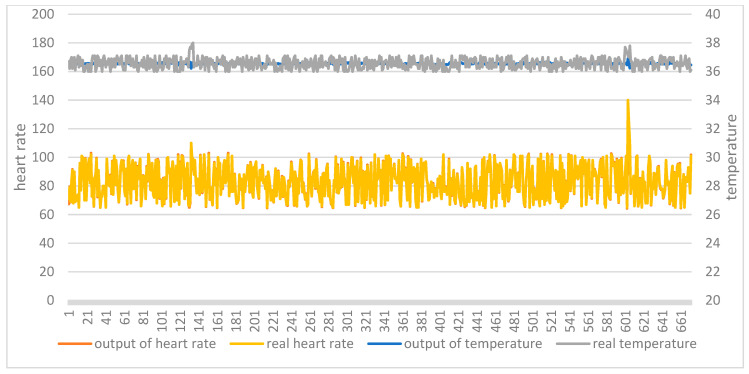
Comparison of input and output of AutoEncoder model in test set 2.

**Figure 14:**
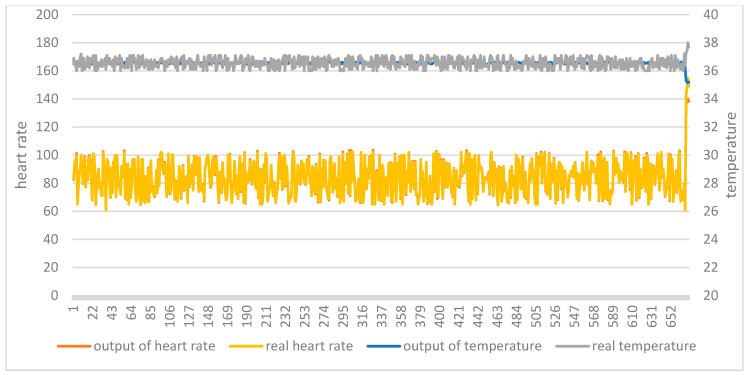
Comparison of input and output of GRU-AE model in test set 1.

**Figure 15:**
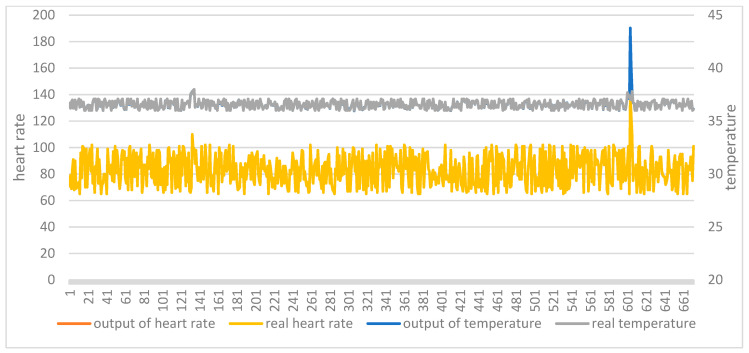
Comparison of input and output of GRU-AE model in test set 2.

The two test sets were then applied to the two trained models. Figure 12 shows the comparison between the original data and the model output of the AutoEncoder model in test set 1. Figure 13 shows the comparison between the original data and the model output of the AutoEncoder model in test set 2. Figure 14 shows the comparison between the original data and the model output of the GRU-AE model in test set 1. Figure 15 shows the comparison between the original data and the model output of the GRU-AE model in test set 2. The abscissa indicates the time sequence of the test set. The ordinate on the left represents heart rate, and the ordinate on the right represents body temperature. From the figures, it is found that the original data of the AutoEncoder model is almost the same as the model output data. It may not be able to monitor unhealthy biomedical signals; while the GRU-AE model has some obvious differences in some data. However, it may also be that healthy data are monitored as unhealthy. In addition, the two models have better auto-encoding effects on most healthy data.

**Figure 16:**
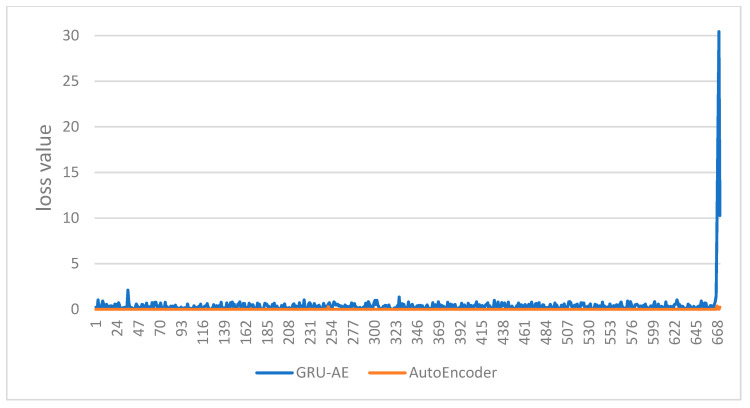
Comparison of the loss value changes of the two models in test set 1.

**Figure 17:**
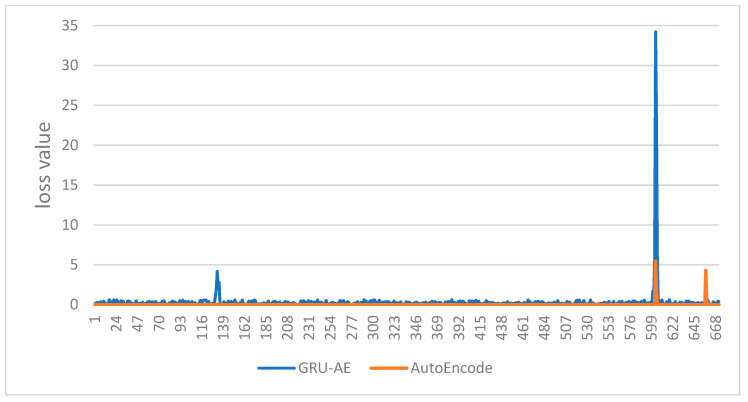
Comparison of the loss value changes of the two models in test set 2.

Whether the monitoring result is healthy or not can be determined by comprehensive consideration of the two biomedical signals. Therefore, the aforementioned original data and output data are processed by loss operation. The final loss values are as shown in the figures. The abscissa indicates the time sequence of the test set. The ordinate represents the loss value between the output data and the original data. Figure 16 is the loss value change of AutoEncoder model and the GRU-AE model in test set 1. Figure 17 is the loss value change of AutoEncoder model and the GRU-AE model in test set 2. The figures show that GRU-AE generates more significant losses than the AutoEncoder model on both of the two test sets. The loss differences are even more significant on test set 2. The result is more intuitive. Therefore, the AutoEncoder model is likely to fail to monitor unhealthy conditions successfully. GRU-AE may mistakenly monitor healthy biomedical signal data as unhealthy but is more sensitive to data. 

To evaluate the model monitoring effect more accurately, we constructed a confusion matrix. The ROC curve describing the relationship between *TPR* and *FRP* is shown in the figures. The area under the ROC curve, *AUC*, can accurately describe the performance of the model.

**Figure 18:**
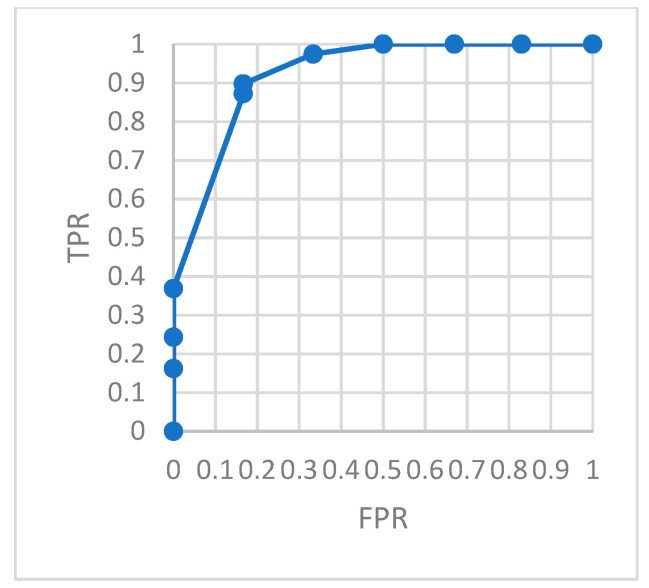
ROC of AutoEncoder in test set 1.

**Figure 19:**
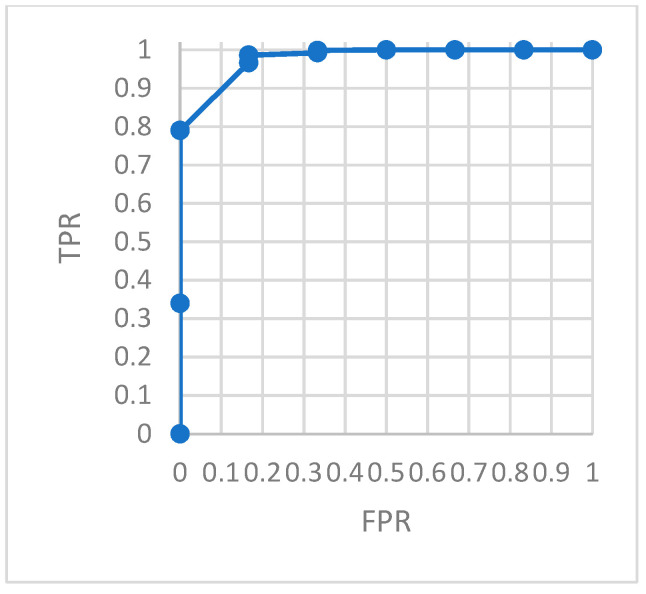
ROC of GRU-AE in test set 1.

**Figure 20:**
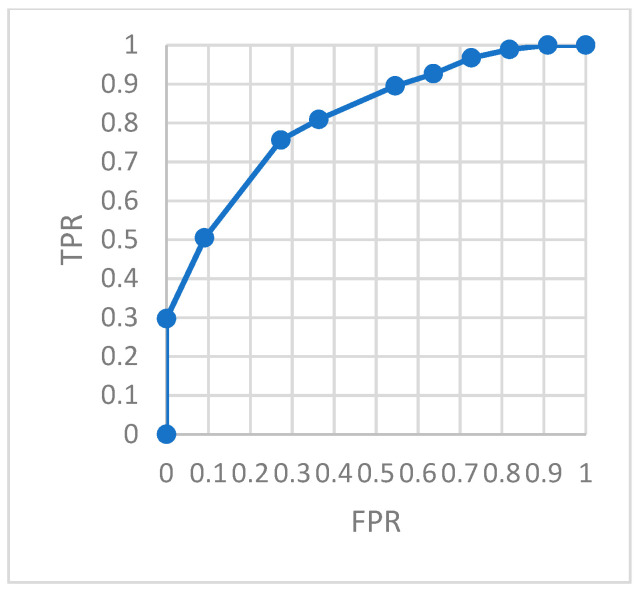
ROC of AutoEncoder in test set 2.

**Figure 21:**
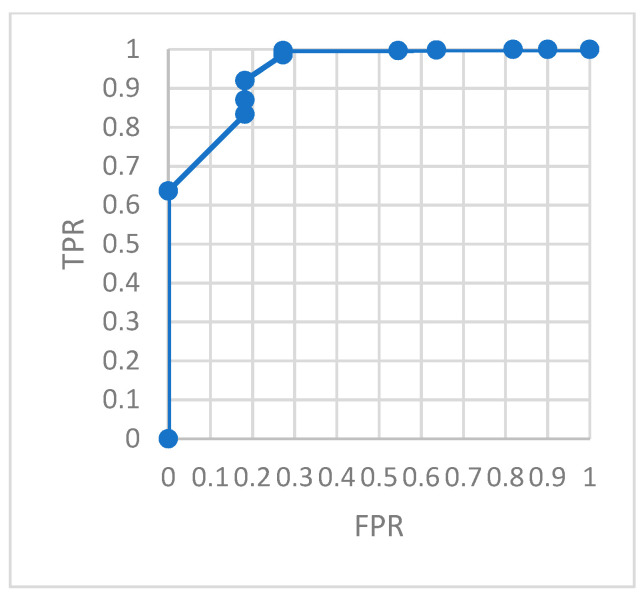
ROC of GRU-AE in test set 2.

Figure 18 is the ROC of the AutoEncoder model in test set 1. Figure 19 is the ROC of the GRU-AE model in test set 1. Figure 20 is the ROC of the AutoEncoder model in test set 2. Figure 21 is the ROC of the GRU-AE model in test set 2. The abscissa of the figure is *FPR*, and the ordinate is *TPR*.

As shown in the figures, the area under the ROC curve of the GRU-AE model is relatively large in both test sets. The classification accuracy is excellent. In the test set 1, the performance is better due to fewer unhealthy biomedical signal data. However, obviously it is better than the AutoEncoder model. In test set 2, the performance of the GRU-AE model is still excellent. According to the calculation, on Test set 1, the *AUC* of the AutoEncoder model is 0.91, and the *AUC* of GRU-AE is 0.978. On Test set 2, the *AUC* of the AutoEncoder model is 0.82, while the *AUC* of GRU-AE is 0.95.

### 5.3. Statistical Analysis

*MAE* and *RMSE* were calculated with the data collected from 2000 subjects. The result of RMSE/MAE was 1.03. The errors of data in this paper are of the same magnitude.

The monitoring accuracy of health issues is an important indicator. The accuracies of health issues and the overall accuracies are shown in Table 2.

**Figure 22:**
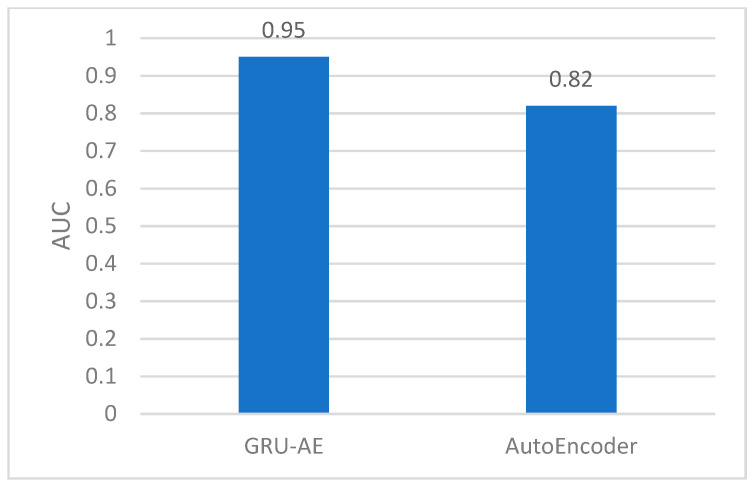
Comparison of *AUC*.

The results in Table 2 show that in 2000 subjects, the total number of subjects with healthy and unhealthy status accurately monitored by us was 1924. Among 613 unhealthy subjects, the number we accurately monitored is 581. The two monitoring results were much better than the comparison method. For remote health monitoring based on biomedical signal data obtained by portable wearable devices, the method proposed in this paper displayed high performance. Furthermore, *AUCs* for the 2000 subjects were calculated for the two models, respectively, according to the above evaluation process. Then the *AUCs* were averaged out. According to the *AUCs*, the corresponding threshold value was obtained. Figure 22 shows the results of *AUC*. It is found that the *AUC* of the GRU-AE model in this paper is 0.95, while that for the traditional AutoEncoder model is only 0.82.

Some existing methods based on deep learning have been widely used in the field of telemedicine [41,42]. In reference [42], they used the medical Korea National Health and Nutrition Examination Survey context data to verify the performance of the CNN-based regular pattern mining model. Although our experimental data did not come from hospitals and other medical institutions, we recorded the biomedical signals of 2000 subjects in 24 weeks of normal life. This is consistent with the telemedical monitoring services provided by various medical institutions. Through the health monitoring experiment, we can infer that the proposed method can be effectively applied to remote health monitoring in telemedicine. If relying on more professional medical monitoring equipment, our monitoring framework and core methods will achieve higher monitoring accuracy under telemedicine scenarios.

### 5.4. Discussion

Summing up, the results show that our proposed GRU-AE deep learning model is able to deal with the issues raised in former studies which are a lack of time series data processing capability and the inability of the fusion analysis of multi-dimensional signal data. As we all know, health monitoring is a complex task. To further improve the accuracy of monitoring results, in addition to improving the existing monitoring model, it is also necessary to incorporate the characteristics of the data and comprehensively analyze the multi-dimensional data from both the horizontal and vertical perspectives. In fact, biomedical data have different time series features and characteristics in healthy and unhealthy states. The time series data are also interconnected within certain context, which needs to be effectively processed, that is, from a vertical perspective. In addition, there is also a certain internal relationship between different biomedical signals (based on a biological perspective), and multi-dimensional biomedical signal data need to be fused and analyzed from a horizontal perspective.

In our remote biomedical signal monitoring framework, we deployed IoT devices, 5G communications and a novel GRU-AE deep learning model to enable the process of time series data and the fusion analysis of the multi-dimensional data. In order to benchmark the performance (the ability to solve the above issues) of our proposed model, we introduced health issues’ overall accuracy and *AUC* metrics. Meanwhile we introduced the ratio of *RMSE* to *MAE* to diagnose the variation in the errors in a set of forecasts. Compared with the traditional AutoEncoder model, shown in Table 2, the monitoring accuracy of health issues was increased by 18.4% and the overall accuracy was increased by 11%. In analyzing the performance on *AUC* metric, the results in Figure 22 indicate that the GRU-AE was superior to the traditional AutoEncoder model (i.e., *AUC* increased by 15.9%). These results reveal that our proposed model significantly improves the accuracy of health monitoring. The increment in accuracy also indicates a better precision in health issue judgement. It can be seen that the improvement of monitoring accuracy can benefit from combining the effective analysis of time series data and multi-dimensional data fusion analysis.

Compared to existing studies, such as the studies of [12,19,22], reference [12] used an IoT-based health monitoring platform to track heart rate and body temperature, and sent email/SMS warnings when these readings were higher than the main value, and no biomedical fusion analysis was implemented. In reference [19], they extracted the time series data of various biological signals separately, and then used CNN to perform abnormality detections separately. No fusion analysis of multi-dimensional signals was considered. In reference [22], they used the MDCA method to fuse a variety of biological signals. However, they did not perform pre/post-correlation analysis based on time series in achieving health monitoring. Our proposed model not only evaluates health from multi-dimensional biomedical signals, but also combines time-series features to realize intelligent monitoring As a consequence, our model achieves more accurate and efficient intelligent health monitoring. The proposed framework uses IoT devices to collect data, while transmitting data based on the 5G network causes latency in data transmission which is not accepted in real-time monitoring applications. In addition, the GRU-AE model proposed in this paper uses GRU units to increase the model’s time series sensitivity, using the AE model to process multi-dimensional biomedical signals and achieve abnormal monitoring. On this basis, the time-related parameters in the model and the fully connected output layer are further optimized, thereby improving the accuracy of the monitoring results. It is especially suitable for the intelligent monitoring of biomedical signals such as body temperature in the COVID-19 pandemic. However, for realizing the application of the framework in different scenarios (e.g., cardiovascular diseases monitoring using ultrasound images), our framework is not suitable for health monitoring of biomedical images. Therefore, a fully fledged remote biomedical signal monitoring framework can process biomedical signal data with different structures, especially with the ability to process unstructured data such as image data. Common image data include ECG, ultrasound images, and so on. We can introduce deep learning models such as AlexNet, VGG19, GoogleNet, and ResNet [43] to process this type of data, and perform fusion analysis with structured biomedical signal data. We believe that after improvements, the health monitoring function will be more systematic and perfect, and applied to more fields.

## 6. Conclusions

This paper proposes a biomedical signal monitoring framework based on deep learning in combination with the IoT, 5G communication, and artificial intelligence technique. The main contributions are summarized as follows:

1. A biomedical signal monitoring framework based on deep learning is proposed. It can collect, transmit and intelligently analyze multi-dimensional biomedical signals in real time to realize remote health intelligent monitoring.
2. A GRU-AE biomedical signal monitoring model is proposed, which addresses the time series biomedical signals data to realize personalized intelligent analysis.
3. The experimental platform of biomedical signal monitoring framework based on deep learning is built. The experiment collected the biomedical signal data of 2000 subjects, and successfully realized remote health monitoring. The experimental results show that GRU-AE outperforms the traditional AutoEncoder model.

Considering the COVID-19 pandemic scenario, remote health monitoring can obtain and analyze human body temperature or other biomedical signals while avoiding direct body contact. Furthermore, it can reduce the risk of transmission and labor costs, and improve monitoring efficiency and accuracy. In future work, this framework will be applied to remote health monitoring of the COVID-19 pandemic, such as quarantining people. In addition, the framework can also carry out intelligent remote health monitoring for the elderly and those in a recovery period.

## Figures and Tables

**Table 1:** The unhealthy biomedical signal in test set.

Test Set 1			Test Set 2		
Time Sequence	Temperature	Heart Rate	Time Sequence	Temperature	Heart Rate
667	37.6	80	131	37.5	66
668	37.4	68	132	37.7	70
669	37.3	162	133	38	110
670	37.7	168	134	37.9	100
671	38	165	135	37.6	99
672	37.7	169	601	37.7	89
			602	37.5	101
			603	37.3	65
			604	37.1	1140
			605	37	125
			606	37.8	110

**Table 2:** Comparison of monitoring accuracy.

	GRE-AE	AutoEncoder
accuracy of health issues	94.8%	80.1%
overall accuracy	96.2%	86.7%

## Data Availability

The data used to support the findings of this study are available from the corresponding author upon request.
